# Fabrication of Z-Type TiN@(A,R)TiO_2_ Plasmonic Photocatalyst with Enhanced Photocatalytic Activity

**DOI:** 10.3390/nano13131984

**Published:** 2023-06-30

**Authors:** Wanting Wang, Yuanting Wu, Long Chen, Chenggang Xu, Changqing Liu, Chengxin Li

**Affiliations:** 1 Shaanxi Key Laboratory of Green Preparation and Functionalization for Inorganic Materials, School of Material Science and Engineering, Shaanxi University of Science & Technology, Xi’an 710021, China; 2 State Key Laboratory for Mechanical Behavior of Materials, School of Materials Science and Engineering, Xi’an Jiaotong University, Xi’an 710049, China

**Keywords:** Z-type system, LSPR, photocatalyst, TiO_2_

## Abstract

Plasmonic effect-enhanced Z-type heterojunction photocatalysts comprise a promising solution to the two fundamental problems of current TiO_2_-based photocatalysis concerning low-charge carrier separation efficiency and low utilization of solar illumination. A plasmonic effect-enhanced TiN@anatase-TiO_2_/rutile-TiO_2_ Z-type heterojunction photocatalyst with the strong interface of the N–O chemical bond was synthesized by hydrothermal oxidation of TiN. The prepared photocatalyst shows desirable visible light absorption and good visible-light-photocatalytic activity. The enhancement in photocatalytic activities contribute to the plasma resonance effect of TiN, the N–O bond-connected charge transfer channel at the TiO_2_/TiN heterointerface, and the synergistically Z-type charge transfer pathway between the anatase TiO_2_ (A-TiO_2_) and rutile TiO_2_ (R-TiO_2_). The optimization study shows that the catalyst with a weight ratio of A-TiO_2_/R-TiO_2_/TiN of approximately 15:1:1 achieved the best visible light photodegradation activity. This work demonstrates the effectiveness of fabricating plasmonic effect-enhanced Z-type heterostructure semiconductor photocatalysts with enhanced visible-light-photocatalytic activities.

## 1. Introduction

Green nanotechnology driven by solar energy has attracted great interest in alleviating the environmental hazards of pesticides, organic dyes, toxic gases, and industrial wastewater [1,2,3,4,5,6]. Since Carey et al. [7,8] used semiconductors to degrade pollutants in 1976, TiO_2_ has proven to be a material that can be used for environmental purification. However, pure TiO_2_ possesses a wide band gap (about 3.2 eV) [9,10]. Due to this limitation, it only responds to UV light and has low solar energy utilization (about 4%). Furthermore, the recombination rate of photogenerated charge carriers generated after TiO_2_ excitation is much higher than that of interfacial charge transfer, resulting in low activity even under UV light [11]. Therefore, promoting solar utilization and charge carrier separation is the key to improving the photocatalytic performance of the catalysts.

Combining the localized surface plasmon resonance (LSPR) effect with semiconductor photocatalysts is a promising method to promote both the charge carrier separation efficiency and the responsive solar illumination range [12]. Till now, most plasmonic photocatalysts relied on noble metal nanostructures (such as Au or Ag) [13]. However, their potential for practical applications is limited due to their rarity, high cost, low thermal stability, and easy dissolution upon the exposure to air or humidity. Thus, novel plasmonic photocatalysts without noble metal components should be developed to overcome these problems.

Recently, TiN has emerged as an attractive competitor in photocatalytic applications due to its plasmonic resonance absorption properties [14]. In addition, the work function of TiN is about 4 eV versus vacuum [15], which is greater than or equal to the electron affinity of most semiconductor metal oxide photocatalysts, including TiO_2_. Therefore, TiN tends to form a favorable energetic alignment to promote hot carrier-enhanced solar energy conversion [16]. Naldoni et al. [17] explored the plasmonic-enhanced TiO_2_ photocatalysts by coupling with TiN, demonstrating that the LSPR effect of the TiN introduced an enhanced photocurrent generation and photocatalytic activity. Fakhouri et al. [18] demonstrated a significant photoactive improvement to bilayered RF magnetron-sputtering TiN/TiO_2_ thin films due to enhanced charge separation at the heterojunction. Clatworthy et al. [19] demonstrated enhanced photocatalytic activity of TiN-TiO_2_ nanoparticle composites and proposed that hot electrons migration can be promoted due to TiO_2_ photovoltage by combining visible light with UV light. However, there is usually a certain lattice mismatch between different semiconductors, therefore constructed heterostructures usually result in large lattice defects and interface resistance [20]. These lattice defects often form the capturing center of photogenerated carriers [21], and the interface resistance would restrict charge transfer and affect their stability [22], thus greatly affecting the efficiency of charge carrier separation. Therefore, a novel plasmonic photocatalyst without noble metal components could be developed if a nanostructured TiN/TiO_2_ composite with good contact could be created. Zhu et al. [23] found that the epitaxial growth of different semiconductors on conductive precursors and the regulation of growth conditions can significantly reduce the interface contact resistance, which can solve the challenge of building heterostructures to obtain high photogenerated charge separation characteristics. Li et al. [24] fabricated a TiN/TiO_2_ plasmonic photocatalyst by in situ growing TiO_2_ on TiN nanoparticles, demonstrating good visible light photocatalytic performance. Furthermore, Zhang et al. [25] significantly reduced the interface resistance and greatly improved their ability to photoelectrochemical decompose water by forming a strong interface contact of the S–O covalent bond at the interface of the Cu_2_S/Fe_2_O_3_ heterostructure. In our previous work [26], the Ti–O–Zr bonded TiO_2_/ZrTiO_4_ heterointerface was constructed by growing ZrTiO_4_ in situ on TiO_2_ to enhance the transport of photogenerated carriers. However, the possibility of forming a chemically bonded TiO_2_/TiN heterostructure and its synergistic enhancement of photocatalytic activities with the LSPR effect of TiN have yet to be explored.

In addition, fabrication of the direct Z-type heterojunction is an alternative strategy to obtain a semiconductor photocatalyst with high performance due to its advantage in charge carrier separation and utilizing the high-redox properties of each component [27]. For direct Z-type heterojunctions with staggered band structure, the photogenerated electrons on lower CB and the holes on the higher VB recombine. Meanwhile, the electrons and holes with stronger redox abilities are retained [28,29]. Thus, the charge carrier separation can be enhanced, and the highest redox potential of the heterojunction can be retained, thus contributing to the promoted photocatalytic activities. In our previous work, we successfully constructed a direct Z-type A-TiO_2_/R-TiO_2_ heterojunction by synergistically mediating oxygen vacancy contents and the band structure of the catalysts through B-doping [30]. However, to our best knowledge, there is no report concerning the possibility of combining TiN plasmonic enhancement with direct Z-type TiO_2_-based heterojunction.

In this study, to solve the two fundamental problems of current TiO_2_-based photocatalysis on low charge carrier separation efficiency and low utilization of solar illumination, we optimized a unique plasmonic effect-enhanced Z-type TiN@A-TiO_2_/R-TiO_2_ photocatalyst with a strong interface of the N–O chemical bond through hydrothermal in situ oxidation of TiN to (A,R)-TiO_2_. In this photocatalyst system, the desirable visible light absorption could be attributed to the LSPR effect of the TiN component. The charge carrier separation efficiency could be enhanced by the Z-type charge transfer mode at the interface of the A-TiO_2_/R-TiO_2_ heterojunction. The obtained TiN@A-TiO_2_/R-TiO_2_ photocatalyst showed a distinct enhancement in visible light absorption, photocurrent generation, and photodegradation activities, demonstrating a simple way to promote the photoactive properties of semiconductor photocatalysts by fabricating plasmonic effect-enhanced Z-type heterostructures.

## 2. Experimental Section

### 2.1. Chemicals

Commercial titanium nitride (TiN, AR) were procured from Aladdin Reagent Co., Ltd., Shanghai, China. Ethanol (C_2_H_5_OH, CP); hydrogen peroxide (H_2_O_2_, 35 wt%, AR), rhodamine B (RhB, AR), and concentrated sulfuric acid (H_2_SO_4_, AR) were procured from the National Reagent company, Beijing, China. All reagents were used as received.

### 2.2. Preparation of the Catalyst

All samples were prepared by a simple hydrothermal process. Firstly, TiN powder was dispersed in 40 mL of deionized water and sonicated for 15 min to obtain TiN suspension. A total of 1 mL of H_2_SO_4_ (1 M) and a certain amount of H_2_O_2_ was added dropwise and stirred for 2 h. The suspension was then hydrothermally treated at 180 °C for 5 h, then washed and dried at 60 °C for 24 h to obtain the target samples. The hydrolysis degree of TiN was determined by the amount of H_2_O_2_ added. In this work, the mass fraction of added H_2_O_2_ is 0%, 0.5%, 1.0%, 2.5%, and 5.0%. The obtained catalysts were labeled as TiN, sample 1 (S1), sample 2 (S2), sample 3 (S3), and sample 4 (S4), respectively.

### 2.3. Characterization Methods

Compositions were recorded on a D/max-2200PC powder X-ray diffraction (XRD), with Cu Kα radiation over a 2θ ranging from 10° to 70°. Morphologies and microstructures were recorded by SEM (FEI Verios 460, Hillsboro, OR, USA), TEM, and HRTEM (FEI Tecnai G2 F20 S-TWIN, Hillsboro, OR, USA). XPS was studied on an X-ray photoelectron spectroscope (XPS, AXIS SUPRA, Manchester, UK) with a monochromatic Al Kα source. Comparing with the standard binding energy of adsorbed carbon (284.6 eV), charge correction was applied after the peak fitting using the CasaXPS analysis software. UV–Vis diffuse reflectance spectra (DRS) were tested on a UV–Vis-NIR spectrophotometer (Cary 5000, Santa Clara, CA, USA). Photoluminescence (PL) spectra were obtained by a fluorescence spectrophotometer (F-4600, Rigaku, Japan) with the excitation source at 345 nm. EPR spectra were conducted by a Bruker A300 spectrometer, during which DMPO was applied using a 300 W Xe lamp as the light source. The FT-IR spectrum (4000–500 cm^−1^) was obtained on Vertex70 Bruker FT-IR Spectroscopy.

Photoelectrochemical analysis was performed on a CHI760D electrochemical workstation equipped with a 300 W Xe lamp and a cut off filter (>420 nm), in which 20 μL of catalyst slurry on an FTO substrate of 2 cm × 2 cm was used as the working electrode. The slurry was prepared by dispersing 5 mg catalyst powder into polyvinylidene difluoride N-methyl pyrrolidone solution (0.5 g, 2 wt%) through ultrasonic vibration. A total of 0.5 M Na_2_SO_4_ solution, platinum, and Ag/AgCl were used as the electrolyte solution, counter, and reference electrode, respectively.

### 2.4. Photocatalytic Performance

The photocatalytic activity of various catalysts was evaluated by RhB photodegradation. Firstly, 30 mL of RhB solution (10 mg/L) was prepared, then 30 mg of catalyst was added under stirring. Then, the above suspension was illuminated under visible light for 120 min, collected, centrifuged, and measured at regular intervals of 30 min. The peak absorbency of the centrifuged RhB solution at 554 nm was applied to analyze its concentration using a UV–Vis spectrophotometer.

## 3. Results and Discussion

### 3.1. Structural Characterization of the Photocatalysts

**Figure 1:**
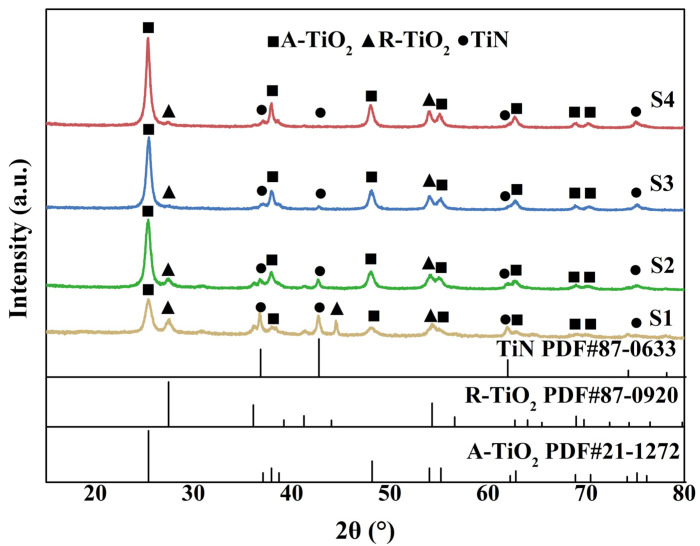
XRD results of the prepared samples obtained with various H_2_O_2_ content.

Figure 1 shows the XRD results of samples obtained with various H_2_O_2_ content. Except for the single-phase TiN sample, all other samples show diffraction peaks ascribed to three phases—that is, A-TiO_2_, R-TiO_2,_ and TiN [31]. Moreover, as the H_2_O_2_ content increases, the peak intensity of TiN decreases and that of TiO_2_ increases. The content of each phase was determined by the Rietveld method and shown in Table 1. With an increase in the H_2_O_2_ content, the phase proportion of TiN gradually decreases accompanied by an increase in the TiO_2_ content, indicating the hydrolysis of TiN and its conversion into TiO_2_. Furthermore, the ratio of A-TiO_2_ to R-TiO_2_ increases accordingly. Specifically, for sample S2, the weight ratio of the three phases (A-TiO_2_:R-TiO_2_:TiN) is about 15:1:1.

**Figure 2:**
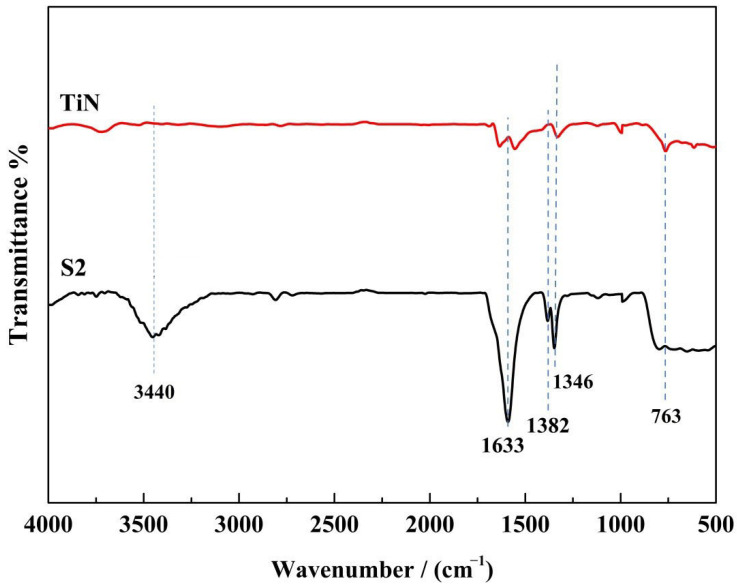
FT-IR spectra of TiN and sample S2.

Figure 2 shows the FT-IR spectra of sample TiN and S2. In the spectra, the wide absorption band at 3440 cm^−1^ and the peaks around 1633 cm^−1^ are ascribed to the adsorbed water and hydroxyl groups [32], respectively. The NO_x_-determined peaks appeared at 1382 cm^−1^ and 1346 cm^−1^ [33]. Furthermore, peaks between 500–800 cm^−1^ are believed to be caused by the stretching vibration of Ti–O–Ti bonds [34]. Compared to the TiN sample, the maximum strength of the Ti–O–Ti bond increased significantly, and a new peak of NO_x_ appeared, indicating the formation of TiO_2_ and the possible existence of a newly formed N–O bond in sample S2.

**Figure 3:**
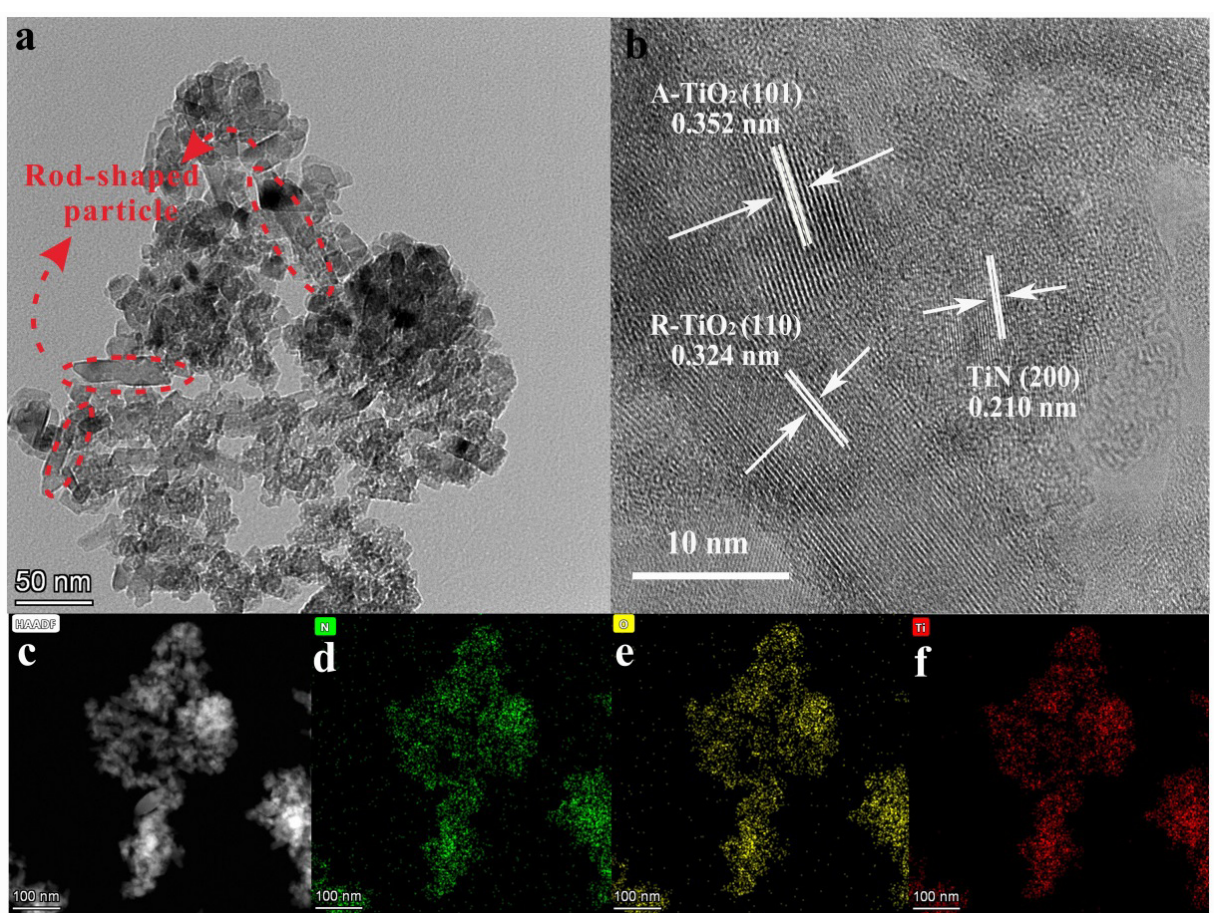
(**a**) TEM; (**b**) HRTEM; (**c**) HADDF images and EDS mappings of the elements N (**d**); O (**e**); Ti (**f**) for S2.

Figures S1–S3 show the SEM images, particle size distributions, and BET surface areas of all the samples. As can be seen, all the samples show uniform and fine particle distribution with an average particle size of about 50 nm, except for sample TiN and S2, which show a slightly smaller size (around 35 nm). Moreover, except for the comparison samples (P25 and TiN), all the other samples have close BET surface areas, suggesting that surface area is not the reason for the photocatalytic performance difference between various samples. Microstructure was studied through TEM analysis. In the TEM results (Figure 3a), uniformly distributed irregular nanoparticles including polygonal, spherical, and rod-shaped particles can be observed. The length of rod particles is 50–200 nm, while the particle size of polygon and spherical particles is about 25 nm. In the HRTEM results (Figure 3b), spacings of 0.210, 0.324, and 0.352 nm of the lattice fringes, correspond to the (200) plane of TiN, the (110) plane of R-TiO_2_, and the (101) plane of A-TO_2_, respectively [35,36]. Moreover, the (A,R)-TiO_2_ are identified on the surface of TiN, and all three phases are in close contact. Furthermore, the energy spectra (Figure 3c–f) show the evenly distributed Ti, N, and O elements, indicating the potential formation of the TiN/(A,R)-TiO_2_ heterointerface. Therefore, it can be deduced that the in situ oxidation of TiN and growth of (A,R)-TiO_2_ could create heterojunctions with an intimate contact interface, improving charge transfer efficiency.

**Figure 4:**
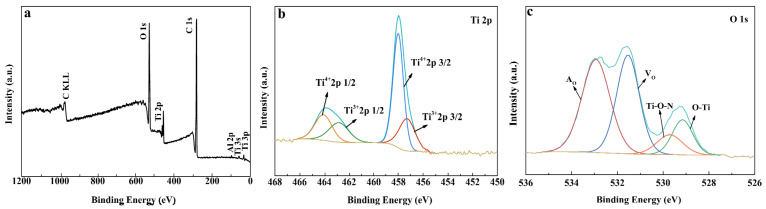
XPS spectra of S2: (**a**) full spectrum; (**b**) Ti 2p; and (**c**) O1s.

Figure 4a is the XPS survey spectrum of S2, demonstrating the presence of C, O, and Ti elements. The N element was not detected, suggesting that it may appear in the interior of the particles. The Ti 2p XPS spectrum was fitted into four peaks. The Ti 2p3/2 (458.1 eV) and the Ti 2p1/2 (464.1 eV) were for TiO_2_ [37,38,39,40]. The Ti 2p3/2 (457.3 eV) and Ti 2p1/2 (462.5 eV) were for partially oxidized TiN [41]. This observation confirms the creation of TiO_2_ from the oxidation of TiN and suggests the possibility of forming a chemical contact interface between TiN and TiO_2_. By further analysis of the O 1s spectrum of S2 (Figure 4c), four peaks can be fitted at 533.09, 531.54, 529.71, and 529.15 eV, which could be attributed to adsorbed H_2_O (A_O_), Ti–O in Ti_2_O_3_ suggests the existence of oxygen vacancies (V_O_), and oxygen in Ti–O–N and Ti–O lattices [42,43,44], respectively. Considering the relatively high area ratio of the Vo XPS peak, it can be deduced that there is a high content of V_O_ in the sample. Based on the analysis of the O1s spectrum, it is clear that N–O bonds exist between TiN and its oxidation products TiO_2_, which contribute to the good contact interface of the formed TiN/TiO_2_ heterostructure.

### 3.2. Photodegradation Performance

**Figure 5:**
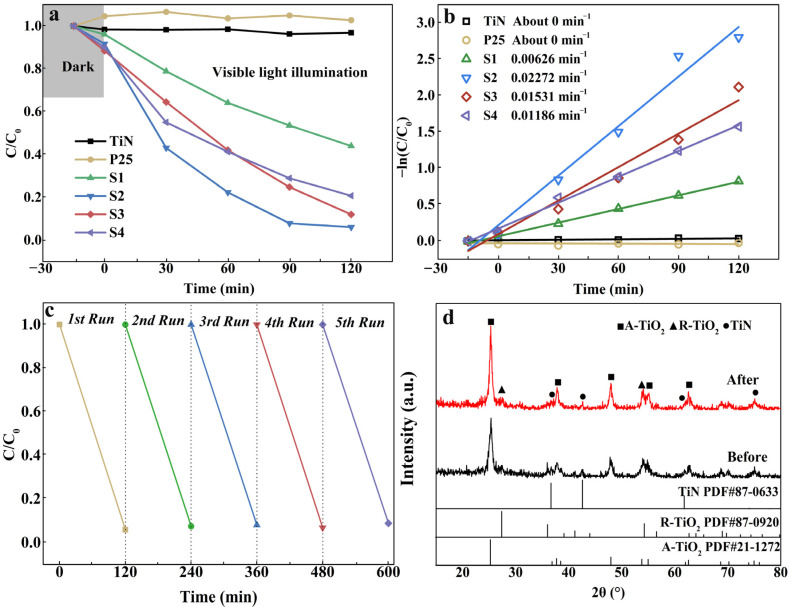
(**a**) Photodegradation performance; (**b**) kinetics of all prepared catalysts; (**c**) cycling experiments of sample S2; and (**d**) XRD patterns of the as-prepared and cycled sample S2.

Figure 5 shows the photodegradation performance of the catalysts. As can be seen, all samples showed no obvious adsorption in the dark reaction. The sample TiN did not have a degrading effect on RhB, indicating that it is not the main catalytic carrier in the photocatalytic reaction process but a cocatalyst. Compared to TiN samples, the hydrolyzed samples showed obvious degrading behavior on RhB. With the deeper degree of hydrolysis, i.e., the decrease in TiN content and the increase in weight ratio of A-TiO_2_ to R-TiO_2_, the photodegradation rate increases first and then decreases. Among them, the S2 sample has the best degradation efficiency, reaching more than 97% in 90 min. As for the kinetics of RhB degradation, the degradation curves are well-fitted by a mono-exponential curve, indicating that the photodegradation experiments follow the first-order kinetics [35]. Figure 5b shows the relationship between ln (C_0_/C) and t for all experiments using different samples, where C_0_ is the initial RhB content and C is the RhB concentration at reaction time t. By regression analysis of the linear curve in the graph, the value of the apparent first-order rate constant can be directly obtained, in which the value of sample S2 is the highest 0.02272 min^−1^. In addition, the cycling experiment (Figure 5c) shows that the sample can maintain a degradation efficiency of more than 90% after five cycles, showing good stability. From the XRD analysis in Figure 5d, no detectable differences can be seen between the as-prepared and cycled S2, indicating a well-preserved crystalline structure of the catalyst after multiple photocatalytic cycles. Moreover, a few studies on the RhB photodegradation performance of TiO_2_-based photocatalysts are summarized in Table 2. The table shows that the visible-light degradation performance of RhB over the catalyst prepared in this work was enhanced, indicating that the prepared TiN@(A,R)TiO_2_ is a promising visible-light photocatalyst.

**Figure 6:**
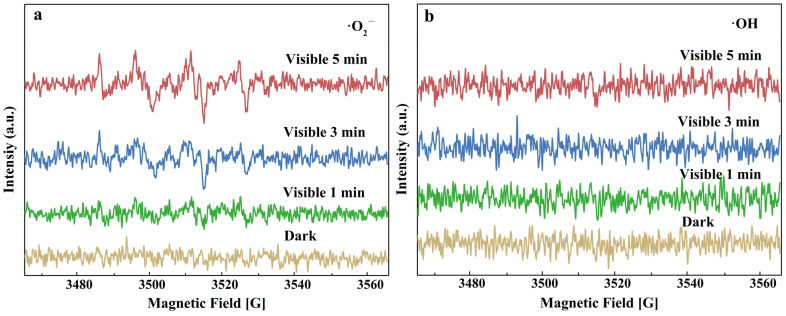
EPR results of (**a**) DMPO-•O_2_^−^; (**b**) DMPO-•OH with sample S2.

Figure S4 shows the result of the free radical capture experiment. By adding IPA, TEOA, BQ, and AgNO_3_ as the capture agents of ·OH, h^+^, ·O_2_^−^, and e^−^, respectively, the effect of free radicals on photocatalysis was investigated [45]. The addition of TEOA and BQ has the greatest impact on the photodegradation rate, suggesting that the corresponding h^+^ and ·O_2_^−^ may play the main role in the photodegradation process. EPR test was carried out on sample S2, and the result is shown in Figure 6. In the O_2_^−^ free radical detection, the DMPO-·O_2_^−^ signal peak of 1:1:1:1 was detected, and its intensity increased with prolonged irradiation time, confirming that the O_2_^−^ radical is the main active species, whereas for ·OH radicals, no DMPO-·OH signal peak of 1:2:2:1 can be detected, suggesting that no ·OH radical can be produced during light irradiation. This result indirectly verified that h^+^ might participate in the following photodegradation reaction without conversion into ·OH.

**Table 2:** Summary of recent relative works on the RhB photodegradation performance of TiO_2_-based heterojunction photocatalysts.

Photocatalyst	C_0_ (mg/L)	Dosage (mg)	Light Source	Degradation Rate	Time (min)	Kinetic Rate (min^−1^)	Ref.
Ag@TiO_2_	10	100	150 W Xe lamp	98.2%	120	0.0188	[46]
TiO_2_ hollow boxes	100	50	Visible light	96.5%	240	0.0025	[47]
Ag_2_O/TiO_2_	4.79	40	UV light	87.7%	80	0.0277	[48]
Ag/ZnO/AgO/TiO_2_	10	30	350 W Xe lamp	99.3%	100	0.0230	[49]
Pt/A/R-TiO_2_	-	-	UV light	92.4%	90	0.0280	[50]
Bi_2_WO_6_/TiO_2_/Pt	20	100	UV light	60.0%	40	0.0210	[51]
g-C_3_N_4/_TiO_2_	50	5	Visible light	87.0%	300	0.0115	[52]
A/R-TiO_2_	10	25	UV light	About 100%	50	-	[53]
Au/A/R-TiO_2_	-	-	UV light	97%	60	0.0470	[54]
TiN@(A,R)TiO_2_	10	30	Visible light	97.0%	90	0.0227	This work

### 3.3. Photocatalytic Mechanism

**Figure 7:**
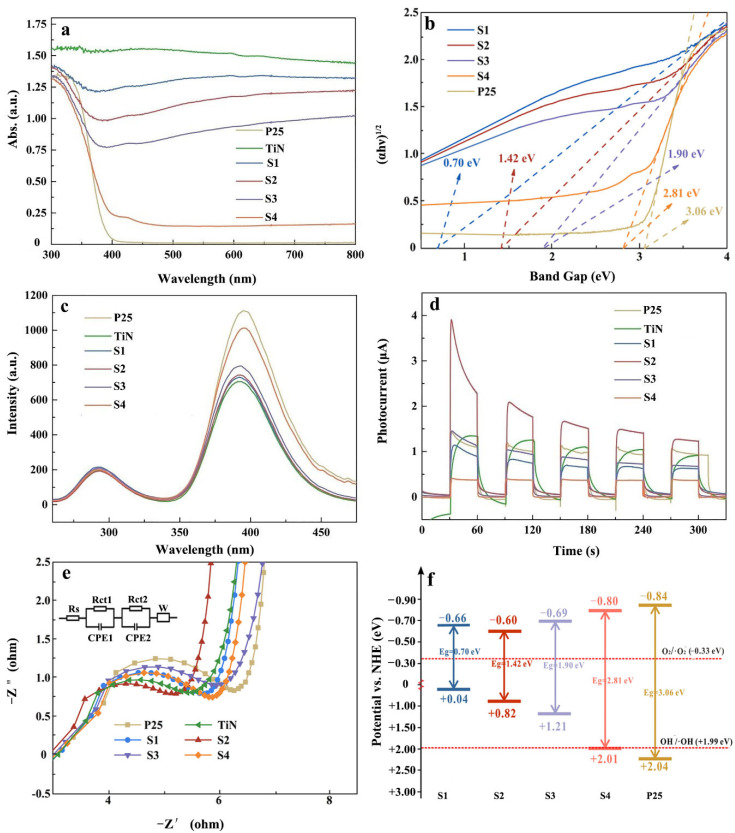
(**a**) UV–Vis DRS; (**b**) Plot of (αhv)^1/2^ versus hν; (**c**) PL spectra; (**d**) TP curves; (**e**) EIS plots; (**f**) band structures of the samples S1 (blue), S2 (red), S3 (purple), S4 (orange), P25 (yellowish-brown) and TiN (green).

Figure 7a shows the UV–Vis DRS spectra of all prepared catalysts. TiN shows full spectrum absorption characteristics similar to those of metals. Compared to P25 and sample S4 with little TiN content showing no obvious visible light absorption, the other samples show obvious light absorption in the entire visible light region (390–780 nm). Moreover, with increasing hydrolysis degree of TiN, the light absorption intensity gradually decreases, confirming that component TiN plays a decisive role in the light absorption ability of the prepared photocatalyst. The result is consistent with the report that the presence of TiN contributed to improving the material’s entire solar light absorption capability [31].

The band gap (Eg) is further obtained through the conversion of Formula (1) [55]:

αhν = A (hν − Eg) ^n⁄2^, (1)

where A is a constant, n = 1 for indirect semiconductors [56], and α and h are the absorption coefficient and photon energy, respectively.

With the decrease in the TiN content, the Eg of the sample gradually increases from 0.70 eV (S1) to 3.06 eV (P25). Therefore, the presence of TiN can effectively reduce the band gap of the sample, thus significantly improving the capability of light absorbance and utilization.

Figure 7c shows the PL spectral analysis of the samples, in which a higher fluorescence intensity represents a higher carrier recombination rate [57]. TiN and P25 showed the lowest and strongest fluorescence intensity, respectively. The fluorescence intensity of the others gradually increased with increasing TiO_2_ phase content. In particular, sample S2 also maintains low fluorescence intensity, indicating its outstanding charge separation ability. From the instantaneous photocurrent results in Figure 7d, the highest transient photocurrent signal can be observed for sample S2. The photocurrent signal decreases remarkably when the TiO_2_ phase content is further increased. The above results demonstrate the photocurrent enhancement effect of TiN on TiO_2_.

Figure 7e shows the electrochemical impedance spectroscopy (EIS) analysis of the samples. In addition, the resultant Nyquist plots (insert in Figure 7e) were fitted with an equivalent circuit using Zman software. As is shown, the equivalent circuit consists of internal resistance (Rs), charge transfer resistance (Rct1, Rct2), Warburg impedance (W), and double-layer capacitance (CPE1, CPE2) [58]. Compared to P25, the charge transfer resistance of the other samples (Figure 7e) is reduced to a certain extent, suggesting that the presence of TiN could improve the conductivity of the samples [59]. In particular, the charge transfer resistance of sample S2 is the lowest, demonstrating the greatest charge transfer rate. The above results demonstrate that the best charge carries separation and transfer can be obtained in sample S2. The reason can be explained by its proper phase proportion, and the formation of the N–O bond at the interface of (A,R)-TiO_2_ and TiN, which can effectively reduce the interface contact resistance of the heterostructure.

The calculated flat band potentials (E_fb_) are shown in Figure S5. The Mott–Schottky curves and calculation process of E_fb_ can be found in Figures S5 and S6. Considering that the E_CB_ of the n-type semiconductor is about 0.1 eV higher than its E_fb_, the E_CB_ of the samples can be further deduced [60]. Combined with the band gap values (Eg), their energy band structures can be obtained (Figure 7f) through the following formula (2) [61]:

E_VB_ = E_CB_ + Eg. (2)

It can be seen in Figure 7f that the presence of TiN can significantly decrease the Eg of the samples by improving the valence band (VB) potential. For sample S2, the Eg was reduced from 3.06 eV to 1.42 eV with the VB position changing from +2.04 to +0.82 eV, and CB position was slightly changed compared to P25. With a narrow band gap, sample S2 is more conducive to generating e^−^ and h^+^ charge carriers, while it shows no ability to produce ·OH active species due to its high valence band position (VB), which is in good agreement with the result of the EPR test.

**Figure 8:**
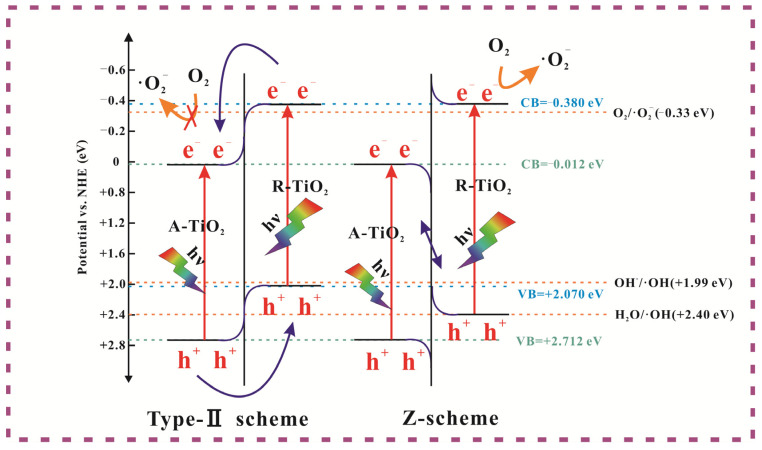
Schematic illustration of the possible charge carrier transfer mode in Type-Ⅱ and direct Z-type photocatalysts.

**Figure 9:**
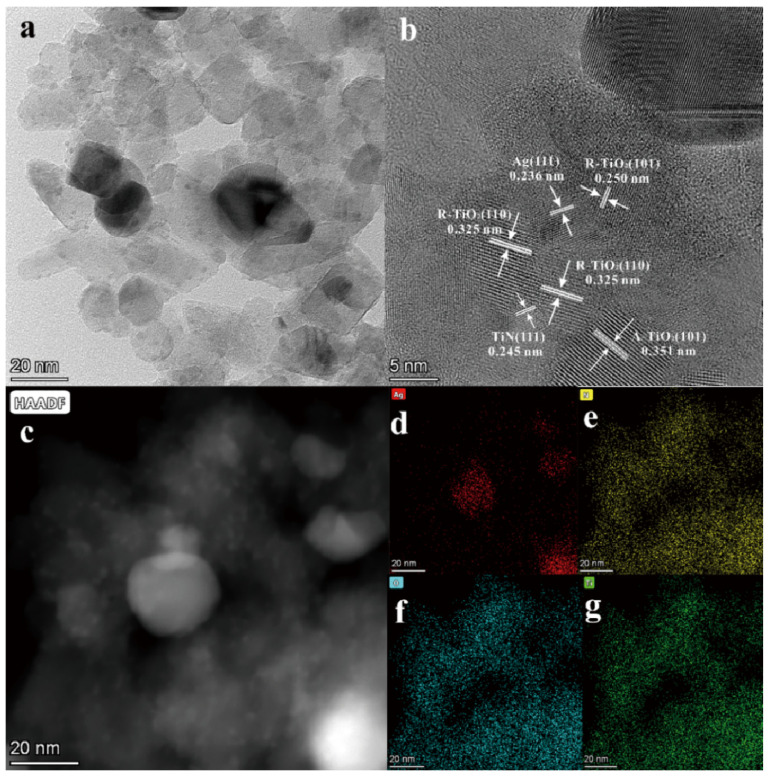
(**a**) TEM; (**b**) HRTEM; (**c**) HADDF images and EDS mappings of the elements Ag (**d**); N (**e**); O (**f**); Ti (**g**) for S2 with photo-deposited Ag nanoparticles.

Considering the staggered band structures of the A-TiO_2_/R-TiO_2_, Type-Ⅱ or Z-type charge transfer modes may occur in the heterojunction, as shown in Figure 8. The values of the CB and VB for (A,R)-TiO_2_ are obtained from the literature [62]. If Type-II mode is formed, electrons transfer to the CB of A-TiO_2_, the reduction potential of which is weak and cannot further reduce surface-adsorbed oxygen to generate ·O_2_^−^ for the following photodegradation process, and this situation is inconsistent with our experimental results. Therefore, the Z-type charge transfer pathway is preferred for the heterojunction constructed in our work. Specifically, according to the literature, for partially reduced samples, the VB and CB positions of R-TiO_2_ are higher than that of A-TiO_2_ and the work function of R-TiO_2_ (φ ≈ 4.3 eV) is smaller than that of A-TiO_2_ (φ ≈ 4.7 eV) [63,64]. When they are in contact, free electrons spontaneously flow from R-TiO_2_ to A-TiO_2_ to obtain their Fermi energy levels to reach equilibrium. At this time, there are a large number of negatively charged electrons near the A-TiO_2_ interface. In contrast, positive charges are gathered at the R-TiO_2_ interface, generating a built-in electric field. Due to the shift in the Fermi energy level, R-TiO_2_ will generate an upward band bending, while A-TiO_2_ will generate a downward band bending [65]. The Z-type electron transfer path is generated due to the formed electric field and the energy band bending. To further prove the formed heterojunction is a Z-type photocatalyst, the Ag nanoparticles were photo-deposited on the catalyst to track where the electrons flow to. Figure 9 shows the EDS, TEM, and HRTEM images of the photodeposition of Ag nanoparticles on sample S2. It shows the uniform distribution of Ag, N, O, and Ti elements, and the Ag nanoparticles were isolated on R-TiO_2_ and apart from A-TiO_2_. The results suggest that the electrons were left on R-TiO_2_, confirming the Z-type charge transfer pathway in the formed heterojunction.

**Figure 10:**
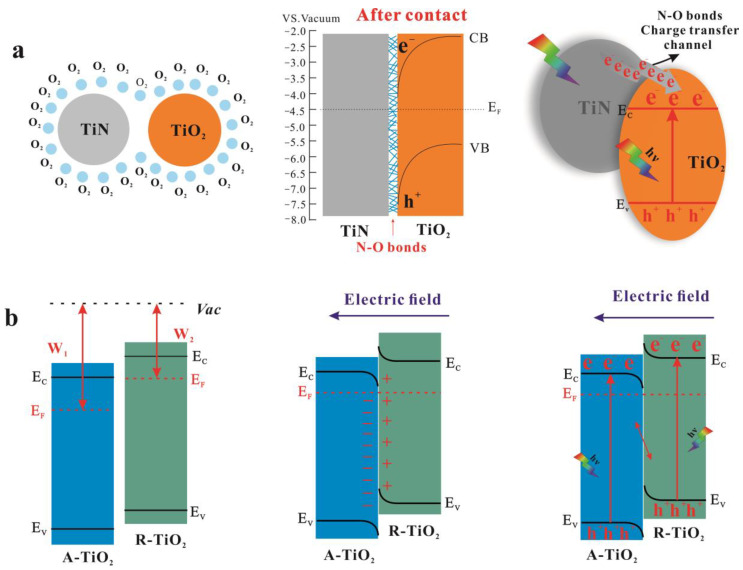
(**a**,**b**) Schematic illustration of the formation and a possible photoinduced catalytic mechanism of the TiN@(A,R)TiO_2_ heterojunction.

In this work, the plasmonic component TiN can broaden the absorbed light range and generate hot electrons due to the LSPR effect. Since nanostructured TiO_2_ was obtained in situ from TiN and charge transfer channel N–O bonds were formed between TiN and TiO_2_, the resulting intimate contacted interface benefits the electron transfer between them. Furthermore, the work function of TiN is ~3.7 eV (φ_m_), and the electron affinity of TiO_2_ is ~4.2 eV (φ_s_). Considering the barrier energy (the lowest energy required for an electron in the metal to be injected into the semiconductor) can be calculated as φ = φ_m_ − φ_s_ [66], a negative value (−0.5 eV) can be obtained, suggesting the quick injecting of the hot electrons into TiO_2_. Therefore, the improved photocatalytic performance of TiN@A-TiO_2_/R-TiO_2_ heterojunction can be concluded and shown in Figure 10. First, the plasmonic properties of TiN greatly broaden the light absorption range, generating and injecting hot electrons into TiO_2_. Furthermore, the N–O bond contacted TiO_2_/TiN heterointerface can significantly reduce the contact resistance of the interface and improve the charge transfer efficiency. Moreover, the optimized three-phase ratio and the formed Z-type A-TiO_2_/R-TiO_2_ heterojunction with an intimate interface contribute to the charge carrier separation and retain its high redox capacity. Thus, more active species will participate in the following photodegradation activities.

## 4. Conclusions

In this work, a plasmonic effect-enhanced TiN@A-TiO_2_/R-TiO_2_ direct Z-type heterojunction was fabricated through the simple hydrothermal reaction process. By regulating the amount of H_2_O_2_ oxidant, the proportion of TiN, anatase TiO_2_, and rutile TiO_2_ contents can be successfully adjusted and the interface charge transfer channel (N–O bond) has been constructed. Due to the Z-type charge transfer path between A-TiO_2_ and R-TiO_2_, the N–O bond connected charge transfer channel at the TiN/TiO_2_ interface, and the synergistic plasma resonance effect of TiN, the optimized photocatalyst shows a distinct increment in visible light absorption, photocurrent generation, and photocatalytic performance, demonstrating an effective approach to promote the photoactive properties of semiconductor photocatalysts.

## Figures and Tables

**Table 1:** Compositions and ratios of the A/R-TiO_2_ and TiN in the prepared samples.

Samples	Content of H_2_O_2_ (wt%)	TiO_2_/wt%			TiN/wt%
		A-TiO_2_	R-TiO_2_	A-TiO_2_:R-TiO_2_	
TiN	0	-	-	-	100%
S1	0.5	80.9%	19.1%	4.25	21.1%
S2	1.0	93.8%	6.2%	15.13	7.2%
S3	2.5	97.3%	2.7%	36.40	1.7%
S4	5.0	98.3%	1.7%	57.82	0.2%

## Data Availability

The data presented in this study are available on request from the corresponding author.

## Supplementary Materials

The following supporting information can be downloaded at: https://www.mdpi.com/article/10.3390/nano13131984/s1, Figure S1: SEM images of (a) TiN, (b) S1, (c) S2, (d) S3, (e) S4. Figure S2: Particle size distribution of all samples (a) TiN, (b) S1, (c) S2, (d) S3, (e) S4. Figure S3: N2 adsorption/desorption isotherm plots and pore size distribution of all samples. Figure S4: Free radical capture experiment of sample S2. Figure S5: Mott-Schottky curves of samples (a) P25, (b) TiN, (c) S1, (d) S2, (e) S3, (f) S4. Figure S6: Schematic diagram of flat band potential of the prepared samples.
