# Supplementary material for: Fabrication of Z-Type TiN@(A,R)TiO2 Plasmonic Photocatalyst with Enhanced Photocatalytic Activity
Source: Nanomaterials (Basel). 2023 Jun 30;13(13):1984. doi: 10.3390/nano13131984 (PMC10343924; doi:10.3390/nano13131984)
Supplement: Supplementary file 1 [file nanomaterials-13-01984-s001.zip › nanomaterials-2426058-supplementary.pdf]

Fabrication of Z-type TiN@(A,R)TiO_2 plasmonic photocatalyst with enhanced photocatalytic activity

Wanting Wang [1], Yuanting Wu [1], Long Chen [1], Chenggang Xu [1], Changqing Liu [2,1*] and Chengxin Li [2]

[1] School of Material Science and Engineering, Shaanxi Key Laboratory of Green Preparation and Functionalization for Inorganic Materials, Shaanxi University of Science & Technology, Xi'an 710021, China

[2] State Key Laboratory for Mechanical Behavior of Materials, School of Materials Science and Engineering, Xi'an Jiaotong University, Xi'an 710049, China

* Correspondence: liu280097311@163.com

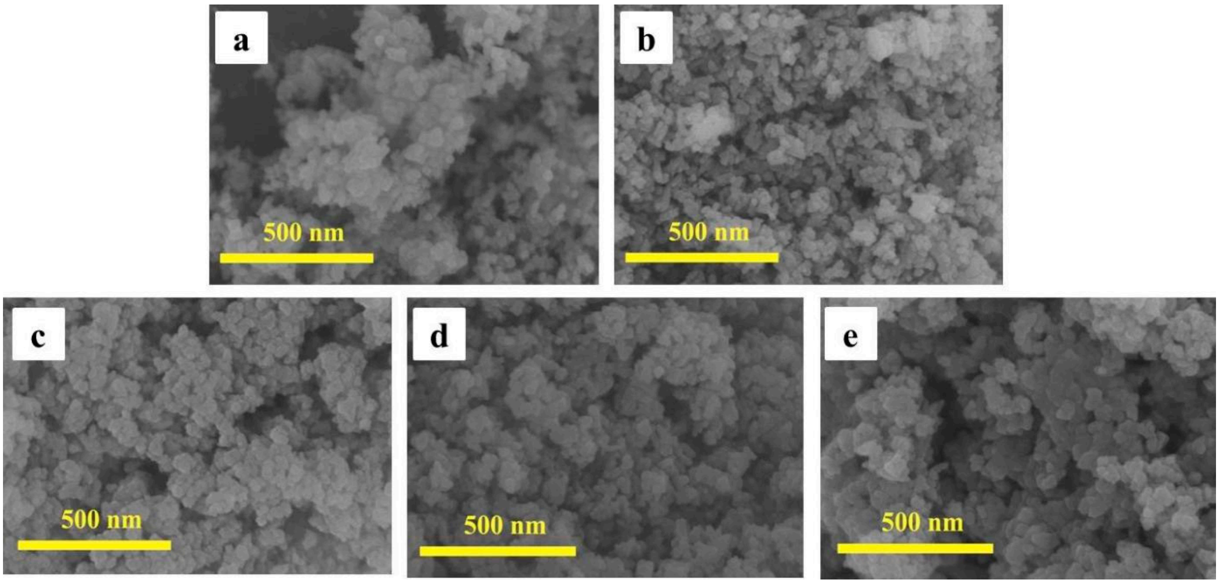

Fig. S1 SEM images of (a) TiN, (b) S1, (c) S2, (d) S3, (e) S4

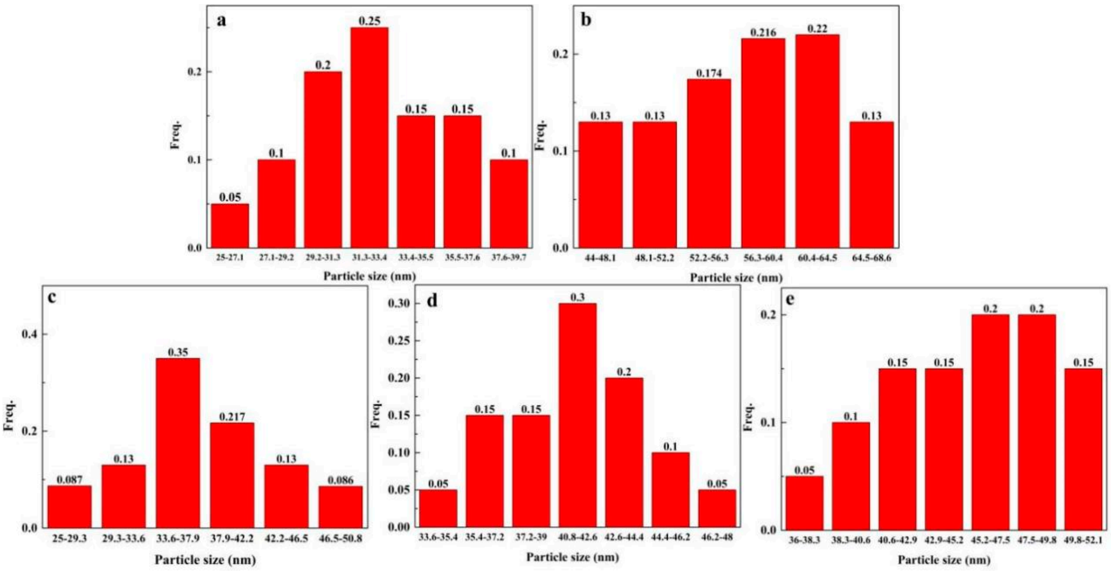

Fig. S2 Particle size distribution of all samples (a) TiN, (b) S1, (c) S2, (d) S3, (e) S4

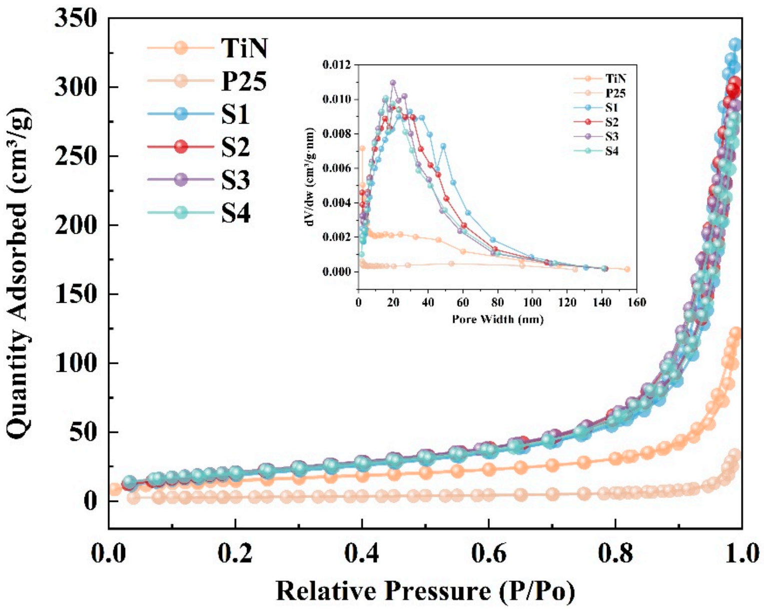

Fig. S3 N_2 adsorption/desorption isotherm plots and pore size distribution of all samples

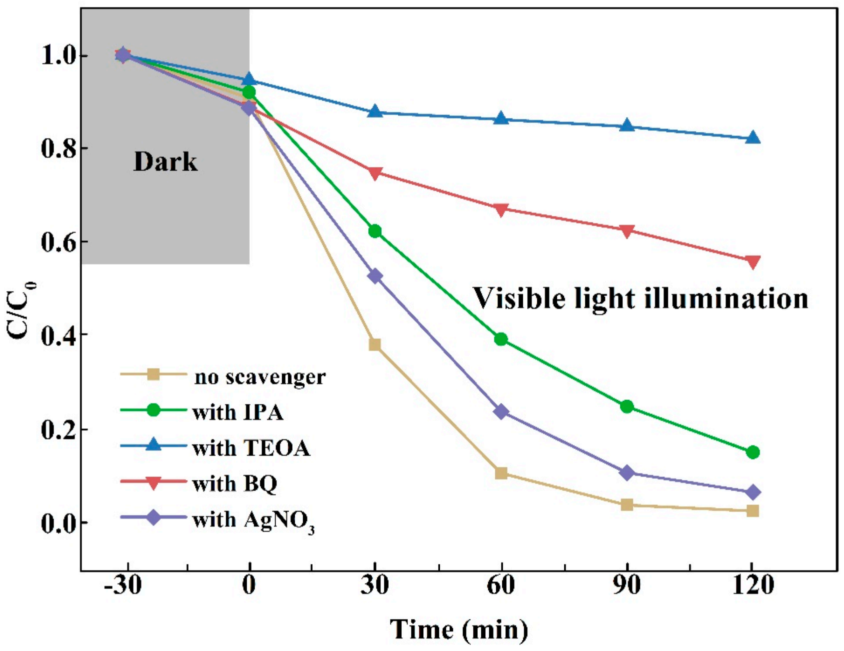

Fig.S4 Free radical capture experiment of sample S2

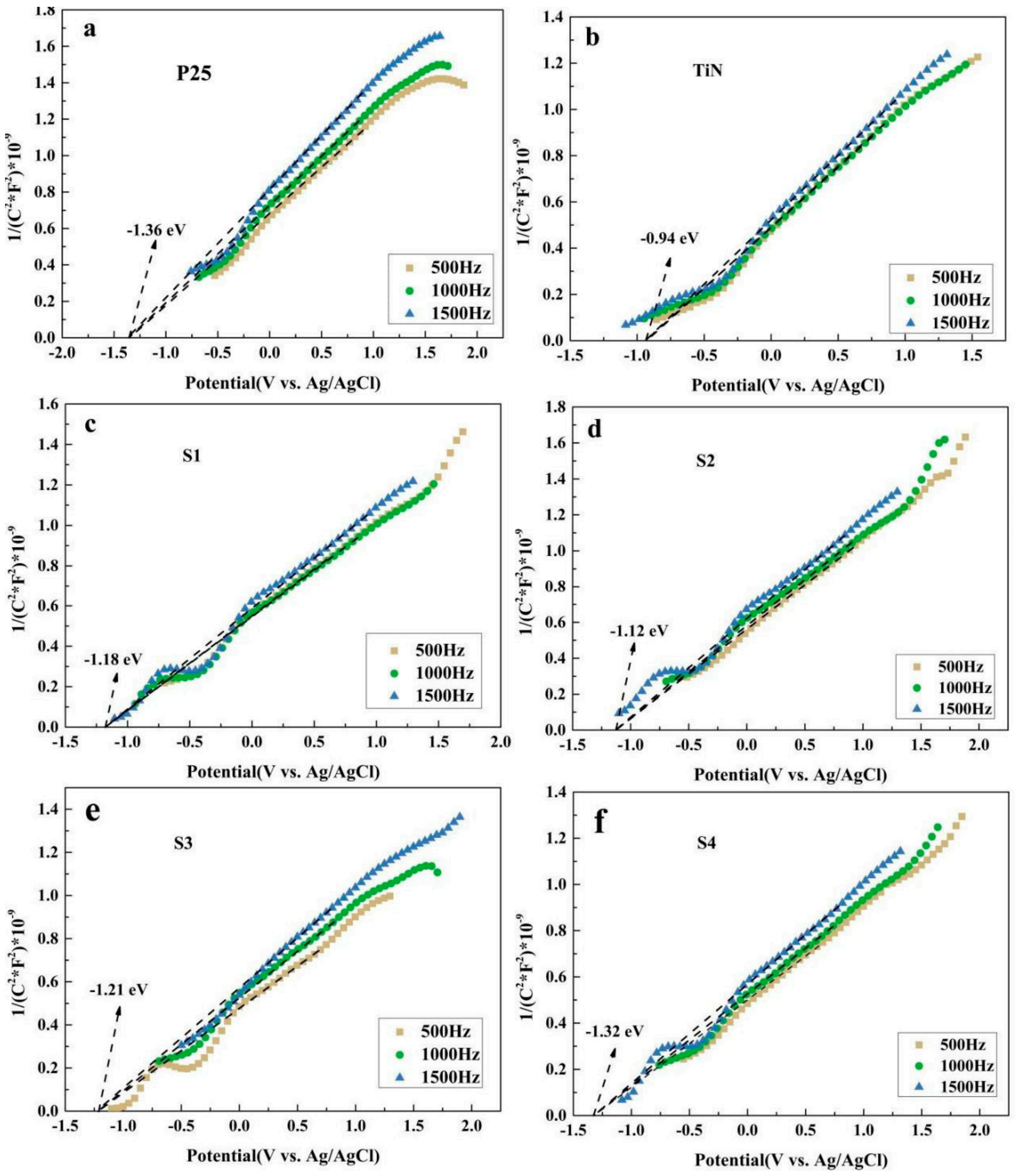

Fig. S5 Mott-Schottky curves of samples (a) P25, (b) TiN, (c) S1, (d) S2, (e) S3, (f) S4

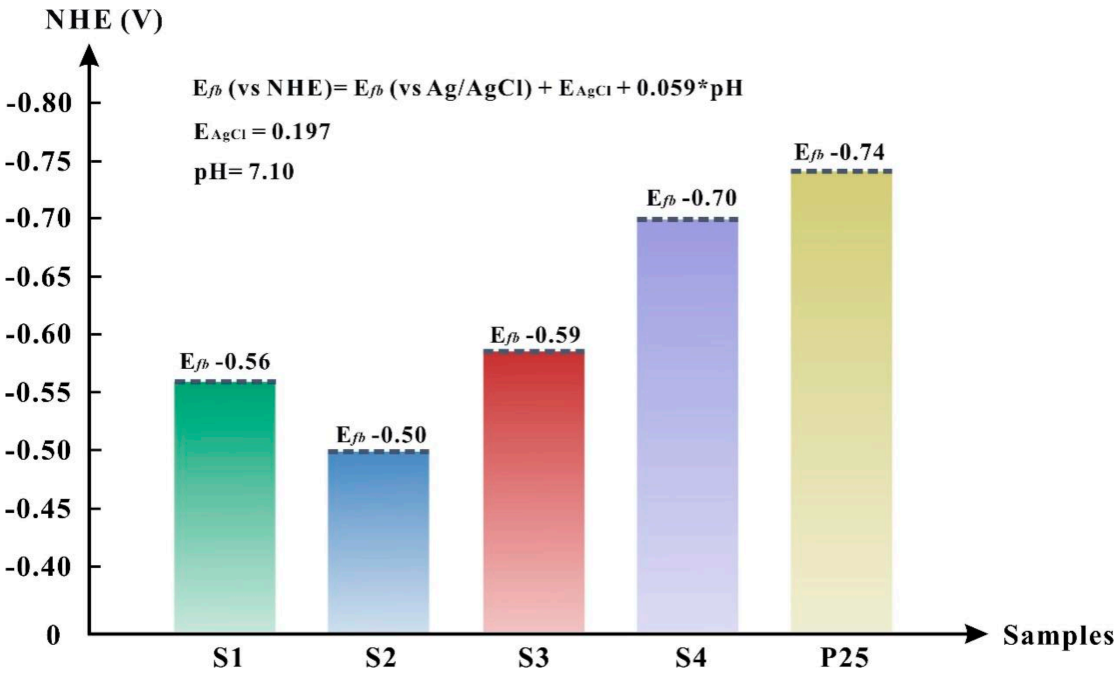

Fig. S6 Schematic diagram of flat band potential of the prepared samples
